# Impact of Translation on Biomedical Information Extraction: Experiment on Real-Life Clinical Notes

**DOI:** 10.2196/49607

**Published:** 2024-04-04

**Authors:** Christel Gérardin, Yuhan Xiong, Perceval Wajsbürt, Fabrice Carrat, Xavier Tannier

**Affiliations:** 1 Institut Pierre Louis d'Epidémiologie et de Santé Publique, Sorbonne Université, Institut National de la Santé et de la Recherche Médicale, Paris, France; 2 Shanghai Jiaotong University, Shanghai, China; 3 Innovation and Data Unit, Assistance Publique Hôpitaux de Paris, Paris, France; 4 Department of Public Health, Assistance Publique Hôpitaux de Paris, Hôpital Saint-Antoine, Paris, France; ^5^Sorbonne Université, Institut National de la Santé et de la Recherche Médicale, Université Sorbonne Paris-Nord, Laboratoire d'Informatique Médicale et de Connaissance en e-Santé, Paris, France

**Keywords:** concept normalization, named entity recognition, natural language processing, translation, translational tool, biomedical data set, bilingual language model

## Abstract

**Background:**

Biomedical natural language processing tasks are best performed with English models, and translation tools have undergone major improvements. On the other hand, building annotated biomedical data sets remains a challenge.

**Objective:**

The aim of our study is to determine whether the use of English tools to extract and normalize French medical concepts based on translations provides comparable performance to that of French models trained on a set of annotated French clinical notes.

**Methods:**

We compared 2 methods: 1 involving French-language models and 1 involving English-language models. For the native French method, the named entity recognition and normalization steps were performed separately. For the translated English method, after the first translation step, we compared a 2-step method and a terminology-oriented method that performs extraction and normalization at the same time. We used French, English, and bilingual annotated data sets to evaluate all stages (named entity recognition, normalization, and translation) of our algorithms.

**Results:**

The native French method outperformed the translated English method, with an overall *F*_1_-score of 0.51 (95% CI 0.47-0.55), compared with 0.39 (95% CI 0.34-0.44) and 0.38 (95% CI 0.36-0.40) for the 2 English methods tested.

**Conclusions:**

Despite recent improvements in translation models, there is a significant difference in performance between the 2 approaches in favor of the native French method, which is more effective on French medical texts, even with few annotated documents.

## Introduction

Named entity recognition (NER) and term normalization are important steps in biomedical natural language processing (NLP). NER is used to extract key information from textual medical reports, and normalization consists of matching a specific term to its formal reference in a shared terminology such as the United Medical Language System (UMLS) Metathesaurus [1]. Major improvements have been made recently in these areas, particularly for English, as a huge amount of data is available in the literature and resources. Modern automatic language processing relies heavily on pretrained language models, which enable efficient semantic representation of texts. The development of algorithms such as transformers [2,3] has led to significant progress in this field.

**Figure 1.**
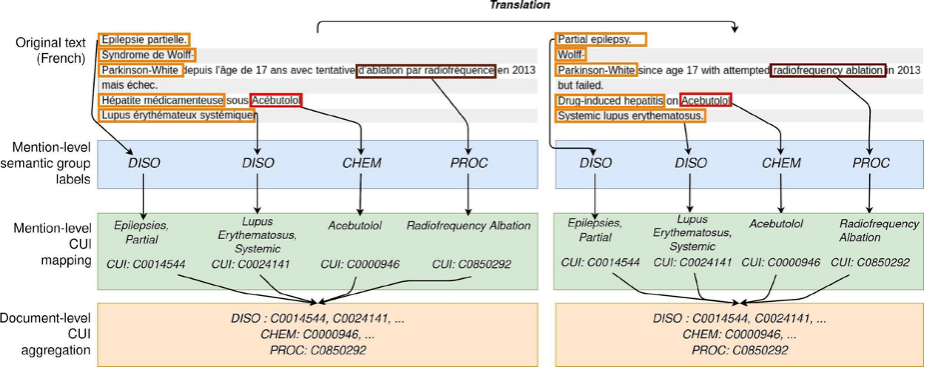
Overall objective of the method: translating plain text to the CUI codes of the UMLS Metathesaurus, document by document. CHEM: Chemicals & Drugs; CUI: concept unique identifier; DISO: Disorders; PROC: Procedures; UMLS: United Medical Language System.

In Figure 1, the term “mention level” indicates that the analysis is carried out at the level of a word or small group of words: first at the NER stage (in blue) and then during normalization (in green); finally, all mentions with normalized concept unique identifiers (CUIs) are aggregated at the “document level” (orange part). The sets of aggregated CUIs per document predicted by the native French and translated English approaches are then compared to the manually annotated gold standard.

In many languages other than English, efforts remain to be made to obtain such results, notably due to a much smaller quantity of accessible data [4]. In this context, our work explores the relevance of a translation step for the recognition and normalization of medical concepts in French biomedical documents. We compared 2 methods: (1) a native French approach where only annotated documents and resources in French are used and (2) a translation-based approach where documents are translated into English, in order to take advantage of existing tools and resources for this language that would allow the extraction of concepts mentioned in unpublished French texts without new training data (zero-shot), as proposed in van Mulligen et al [5].

We evaluated and discussed the results on several French biomedical corpora, including a new set of 42 annotated hospitalization reports with 4 entity groups. We evaluated the normalization task at the document level, in order to avoid a cross-language alignment step at evaluation time, which would add a potential level of error and thus make the results more difficult to interpret (see word alignment in Gao and Vogel [6] and Vogel et al [7]). This normalization was carried out by mapping all terms to their CUI in the UMLS Metathesaurus [1]. Figure 1 summarizes these various stages, from the raw French text and the translated English text to the aggregation and comparison of CUIs at the document level. Our code is available on GitHub [8].

The various stages of our algorithms rely heavily on transformers language models [2]. These models currently represent the state of the art for many NLP tasks, such as machine translation, NER, classification, and text normalization (also known as entity binding). Once trained, these models can represent any specific language, such as biomedical or legal. The power of these models comes from their neural architecture but also largely depends on the amount of data they are trained on. In the biomedical field, 2 main types of data are available: public articles (eg PubMed) and clinical electronic medical record databases (eg MIMIC-III [9]), and the most powerful models are, for example, BioBERT [10], which has been trained on the whole of PubMed in English, and ClinicalBERT [11], which has been trained on PubMed and MIMIC-III. In French, the variety of models is less extensive, with CamemBERT [12] and FlauBERT [13] for the general domain and no specific model available for the biomedical domain.

**Figure 2.**
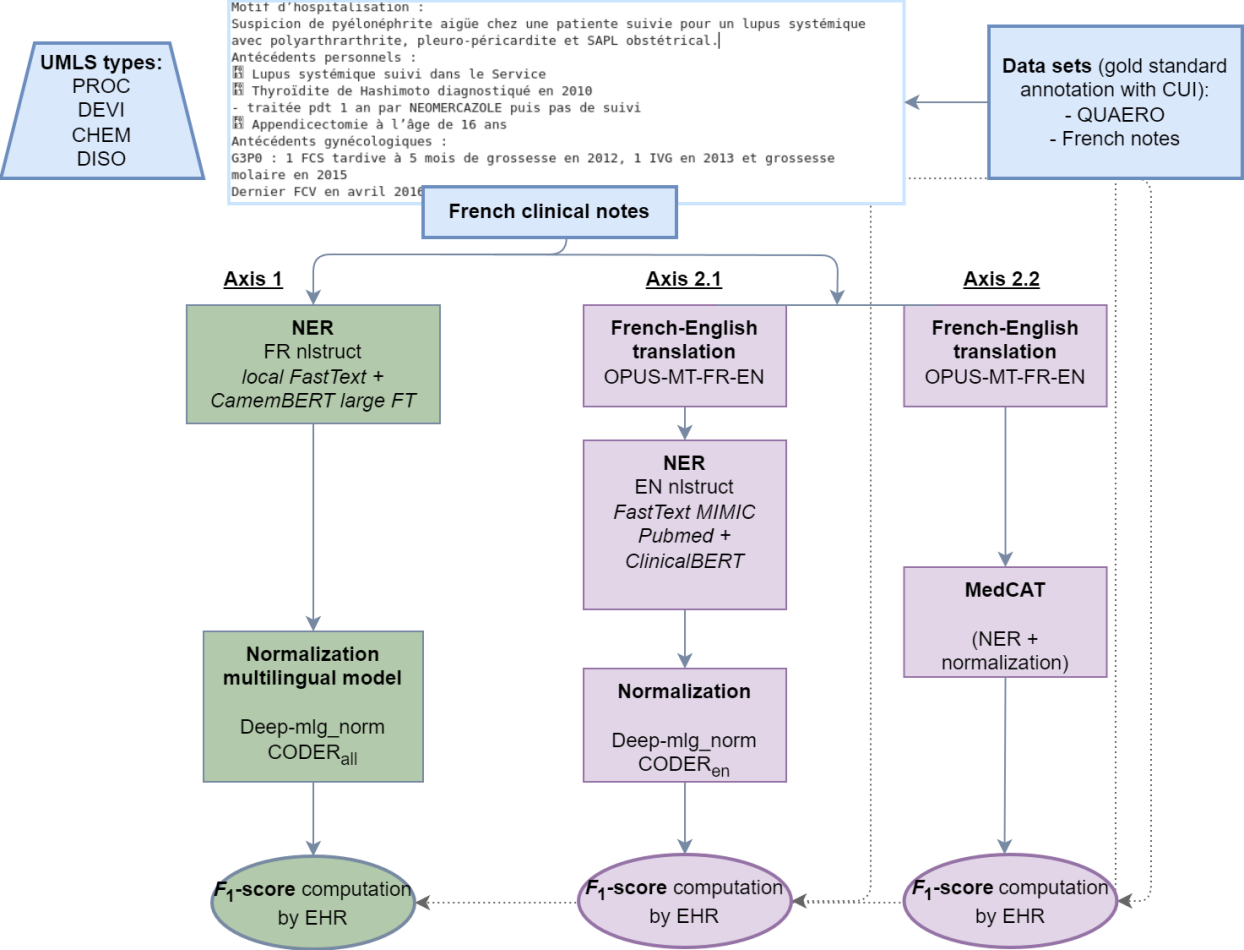
Diagram of different experiments comparing French and English language models without and with intermediate translation steps. CHEM: Chemicals & Drugs; CUI: concept unique identifier; DEVI: Devices; DISO: Disorders; EHR: electronic health record; EN: English; FR: French; FT: fine-tuned; PROC: Procedures; UMLS: United Medical Language System.

In Figure 2, axis 1 (green axis on the left) corresponds to the native French branch with a NER step based on a FastText model trained from scratch on French clinical notes and a CamemBERT model. A multilingual Bidirectional Encoder Representations From Transformers (BERT) model was then used for the normalization step, with 2 models tested: a deep multilingual normalization model [14] and CODER [15] with the full version. Axes 2.1 and 2.2 (the 2 purple axes on the right) correspond to the translated English branches, with a first translation step performed by the OPUS-MT-FR-EN model [16] for both. Axis 2.1 (left) was conducted with decoupled NER and normalization steps: FastText trained from PubMed and MIMIC-III [17] for NER, and deep multilingual normalization [14] or CODER [15] with the English version for normalization. Axis 2.2 (right) used a single system for the NER and normalization stages: MedCAT [18].

In addition to particularly powerful English-language pretrained models, universal biomedical terminologies (ie, the UMLS Metathesaurus) also contain many more English terms than other languages. For example, the UMLS Metathesaurus [1] contains at least 10 times more English terms than French terms, which may enable rule-based models to perform better in English. As mentioned above, each reference concept in the UMLS Metathesaurus [1] is assigned a CUI, associated with a set of synonyms, possibly in several languages, and a semantic group, such as *Disorders*, *Chemicals & Drugs*, *Procedure*, *Anatomy*, etc.

In parallel, the performance of machine translation has also improved thanks to the same type of transformer-based language models, and recent years have seen the emergence of high-quality machine translations, such as OPUS-MT developed by Tiedemann et al [16], Google Translate, and others. These 2 observations have led several research teams to add a translation step in order to analyze medical texts, for example, to extract relevant mentions in ultrasound reports [19,20] or in the case of the standardization of medical concepts [14,15,21]. Work in the general (nonmedical) domain has also focused on alignment between named entities in parallel bilingual texts [22,23].

## Methods

### Approaches

#### Overview

Figure 2 shows the main approaches and models used in our study. We explored 1 “native French approach axis” (axis 1 in Figure 2), based on French linguistic models learned from and applied to French annotated data, and 2 “translated English approach axes” (axes 2.1 and 2.2), based on a translation step and concept extraction tools in English. We compared the performance of all axes with the average of the document-level CUI prediction precisions for all documents.

#### Native French Approach

Axis 1 consisted of 2 stages: a NER stage and a normalization stage. For the NER stage, we used the nested NER algorithm. Next, a normalization step was performed by 2 different algorithms: a deep multilingual normalization model [14] and CODER [15] with the *CODER all* version.

#### Translated-English Approach

First, axes 2.1 and 2.2 consisted of a translation step, performed by the state-of-the-art OPUS-MT-FR-EN [16] or Google Translate algorithm. Second, similar to axis 1, axis 2.1 was based on a NER step and a normalization step. The NER step was performed by the same algorithm but trained on the National NLP Clinical Challenges (N2C2) 2019 data set [24] without manual annotation realignment; for the normalization step, we used the same deep multilingual algorithm [14] and the English version of CODER [15] based on a BioBERT model [10]. This axis allows us to compare 2 methods whose difference lies solely in the translation step.

Axis 2.2 was based on the MedCAT [18] algorithm, which performs NER and normalization simultaneously. In this case, we compared the native French method with a state-of-the-art, ready-to-use English system, which is not available in French.

### Data Sets

For all our experiments, we chose to focus on 4 semantic groups of the UMLS Metathesaurus [1]: *Chemical & Drugs* (“CHEM”); *Devices* (“DEVI”), corresponding to medical devices such as pacemakers, catheters, etc; *Disorders* (“DISO”), corresponding to all signs, symptoms, results (eg, positive or negative results of biological tests), and diseases; and *Procedures* (“PROC”), corresponding to all diagnostic and therapeutic procedures such as imaging, biological tests, operative procedures, etc, as well as the corresponding number of documents.

Table 1 shows the data sets used for all our experiments and the corresponding number of documents. First, 2 French data sets were used for the final evaluation, as well as for training the axis-1 models. QUAERO is a freely available corpus [25] based on pharmacological notes with 2 subcorpora: MEDLINE (short sentences from PubMed abstracts) and EMEA (drug package inserts). We also annotated a new data set of real-life clinical notes from the Assistance Publique Hôpitaux de Paris data warehouse, described in Section S1 in Multimedia Appendix 1.

**Table 1.** Overview of all data sets used. When a data set is used for both training and testing, 80% of the data set is used for training and 20% is used for testing. Thus, for the EMEA data set, 30 documents were used for training and 8 for testing, 34 French notes were used for training and 8 for testing, and so on.

Variables		Languages and data sets						
		French			English		English and French	
		QUAERO [25]		French notes	N2C2^a^ 2019 [24]	Mantra [26]	WMT^b^ 2016 [27]	WMT 2019 [28]
		EMEA	MEDLINE					
Type		Drug notices	MEDLINE titles	French notes	English notes	Drug notices and MEDLINE titles	PubMed abstracts	PubMed abstracts
Size (documents), n		38	2514	42	100	200	>600,000 sent	6542
**Use**								
	Train NER^c^	✓	✓	✓	✓			
	Test NER	✓	✓	✓	✓			
	Normalization	✓	✓	✓	✓			
	Test MedCAT				✓	✓		
	Translation (fine-tuning)						✓	✓
	Translation (test)						✓	

a N2C2: National Natural Language Processing Clinical Challenges.

b WMT: Workshop on Machine Translation.

c NER: named entity recognition.

Second, we used the N2C2 2019 corpus [24] with annotated CUIs, on which we automatically added semantic group information from the UMLS Metathesaurus [1], to train the axis-2.1 system and evaluate the NER and English normalization algorithms. We also used the Mantra data set [26], a multilingual reference corpus for biomedical concept recognition.

Finally, we refined and tested the translation algorithms on the Workshop on Machine Translation biomedical corpora of 2016 [27] and 2019 [28]. A detailed description of the number of respective entities in the data sets can be found in Table S1 in Multimedia Appendix 1.

The annotation methods for the French corpus are detailed in Section S1 and Figure S1 in Multimedia Appendix 1. The distribution of entities for this annotation is detailed in Table S1 in Multimedia Appendix 1.

### Translation

We used and compared 2 main algorithms for the translation step: the OPUS-MT-FR-EN model [16], which we tested without and with *fine-tuning* on the 2 biomedical translation corpora of 2016 and 2019 [27,28], and Google Translate as a comparison model.

### NER Algorithm

For this step, we used the algorithm of Wajsbürt [29] described in Gérardin et al [30]. This model is based on the representation of a BERT transformer [3] and calculates the scores of all possible concepts to be predicted in the text. The extracted concepts are delimited by 3 values: start, end, and label. More precisely, the encoding of the text corresponds to the last 4 layers of BERT, FastText integration, and a max-pool Char-CNN [31] representation of the word. The decoding step is then performed by a 3-layer long short-term memory [32] with learning weights [33], similar to the method in Yu et al [34]. A sigmoid function was added to the vertex. Values (start, end, and label) with a score greater than 0.5 were retained for prediction. The loss function was a binary cross-entropy, and we used the Adam optimizer [35].

In our experiments, for the native French axis (axis 1 in Figure 2), the pretrained embeddings used to train the model were based on a FastText model [36], trained from scratch on 5 gigabytes of clinical text, and a CamemBERT-large model [12] *fine-tuned* on this same data set. For English axis 2.1, the pretrained models were BioWordVec [17] and ClinicalBERT [11].

### Normalization Algorithms

#### Overview

This stage of our experiments was essential for comparing a method in native French and one translated into English, and it consisted of matching each mention extracted from the text to its associated CUI in the UMLS Metathesaurus [1]. We compared 3 models for this step, described below: the deep multilingual normalization algorithm developed by Wajsbürt et al [14]; CODER [15]; and the MedCAT [18] model, which performs both NER and normalization.

These 3 models require no training data set other than the UMLS Metathesaurus.

#### Deep Multilingual Normalization

This algorithm by Wajsbürt et al [14] considers the normalization task as a highly multiclass classification problem with cosine similarity and a softmax function as the last layer. The model is based on contextual integration, using the pretrained multilingual BERT model [3], and works in 2 steps. In the first step, the BERT model is fine-tuned and the French UMLS terms and their corresponding English synonyms are learned. Then, in the second step, the BERT model is frozen and the representation of all English-only terms (ie, those present only in English in the UMLS Metathesaurus [1]) is learned. The same training is used for the native French and translated English approaches. This model was trained with the 2021 version of the UMLS Metathesaurus [1], corresponding to the version used for annotating the French corpus. The model was thus trained on over 4 million concepts corresponding to 2 million CUIs.

#### CODER

The CODER algorithm [15] was developed by contrastive learning on the basis of the medical knowledge graph of the UMLS Metathesaurus [1], with concept similarities being calculated from the representation of terms and relations in this knowledge graph. Contrastive learning is used to learn embeddings through multisimilarity loss [37]. The authors have developed 2 versions: a multilingual version based on the multilingual BERT [3] and an English version based on the pretrained BioBERT model [10]. We used the multilingual version for axis 1 (native French approach) and the English version for axis 2.1. Both types of this model (*CODER all* and *CODER en*) were trained with the 2020 version of UMLS (publicly available models). *CODER all* [15] was trained on over 4 million concepts corresponding to 2 million CUIs, and *CODER en* was trained on over 3 million terms and 2 million CUIs.

For the deep multilingual model and the CODER model, in order to improve performance in terms of accuracy, we chose to add semantic group information (ie, *Chemical & Drugs*, *Devices*, *Disorders*, and *Procedures*) to the model output: that is, from the first *k* CUIs chosen from a mention, we selected the first from the corresponding group.

The MedCAT algorithm is described in detail in Section S1 in Multimedia Appendix 1.

### Ethical Considerations

The study and its experimental protocol were approved by the Assistance Publique Hôpitaux de Paris Scientific and Ethical Committee (IRB00011591, decision CSE 20-0093). Patients were informed that their electronic health record information could be reused after an anonymization process, and those who objected to the reuse of their data were excluded. All methods were applied in accordance with the relevant guidelines (*Commission nationale de l'informatique et des libertés* reference methodology MR-004 [38]).

## Results

The sections below present the performance results for each stage. The N2C2 2019 challenge corpus [24] enabled us to evaluate the performance of our English models on clinical data, and the Biomedical Translation 2016 shared task [27] allowed us to evaluate our translation performance on biomedical data with a BLEU score [39].

### NER Performances

To be able to compare our approaches in native French and translated English, we used the same NER model, trained and tested on each of the data sets described above. Table 2 shows the corresponding results. Overall *F*_1_-scores were similar across data sets: from 0.72 to 0.77.

**Table 2.** Named entity recognition (NER) performance for each model. For all experiments, we used the same NER algorithm but with different pretrained models. The best performance values are italicized.

Groups	Data sets and models								
	EMEA test, with FastText*^a^ and CamemBERT-FT [12]			French notes, with FastText* and CamemBERT-FT			N2C2^b^ 2019 test, with BioWordVec [17] and ClinicalBERT [11]		
										Precision	Recall	*F*_1_-score	Precision	Recall	*F*_1_-score	Precision	Recall	*F*_1_-score
CHEM^c^	0.80	0.83	0.82	0.84	0.88	0.86	0.87	0.85	0.86
DEVI^d^	0.42	0.81	0.55	0.00	0.00	0.00	0.58	0.51	0.54
DISO^e^	0.54	0.63	0.59	0.67	0.65	0.66	0.74	0.72	0.73
PROC^f^	0.73	0.78	0.74	0.78	0.72	0.75	0.80	0.78	0.79
*Overall*	*0.71*	*0.77*	*0.74*	*0.73*	*0.71*	*0.72*	*0.78*	*0.76*	*0.77*

a FastText* corresponds to a FastText model [36] trained from scratch on our clinical data set.

b N2C2: National Natural Language Processing Clinical Challenges.

c CHEM: Chemical & Drugs.

d DEVI: Devices.

e DISO: Disorders.

f PROC: Procedures.

### Normalization Performances

This section presents only the normalization performance based on the gold standard’s entity mentions, without the intermediate steps. The results are summarized in Table 3. The deep multilingual algorithm performed better for all corpora tested, with an improvement in *F*_1_-score from +0.6 to +0.11. By way of comparison, the winning team of the 2019 N2C2 had achieved an accuracy of 0.85 using the N2C2 data set directly to train their algorithm [24]. In our context of comparing algorithms between 2 languages, the normalization algorithms were not trained on data other than the UMLS Metathesaurus. MedCAT’s performance (shown in Table S2 in Multimedia Appendix 1) cannot be directly compared with that of other models, as this method performed both NER and normalization in a single step. However, we note that this algorithm performed as well as axis 2.1 in terms of overall performance, as shown in Table 4.

**Table 3.** Performance of the normalization step. Model results were calculated from the annotated data sets, focusing on the 4 semantic groups of interest: *Chemical & Drugs*, *Devices*, *Disorders*, and *Procedures*. The best performance values are italicized.

Algorithms	Data set models		
	EMEA test	French notes	N2C2^a^ 2019 test
Deep multilingual normalization	*0.65*	*0.57*	*0.74*
CODER all	0.58	0.51	—^b^
CODER en	—	—	0.63

a N2C2: National Natural Language Processing Clinical Challenges.

b Not applicable.

**Table 4.** Overall performances. The normalization step was performed by the deep multilingual model and the translation was performed by the OPUS-MT-FR-EN FT model. The best performance values are italicized.

Methods	EMEA test			French notes		
	Precision	Recall	*F*_1_-score (95% CI)	Precision	Recall	*F*_1_-score (95% CI)
Axis 1 (French NER^a^+normalization)	0.63	0.60	*0.61 (0.53-0.65)*	0.49	0.53	*0.51 (0.47-0.55)*
Axis 2.1 (Translation+NER+normalization)	0.53	0.40	0.45 (0.38-0.51)	0.41	0.38	0.39 (0.34-0.44)
Axis 2.2 (Translation+MedCAT [18])	0.53	0.46	0.49 (0.38-0.54)	0.38	0.38	0.38 (0.36-0.40)

a NER: named entity recognition.

### Translation Performances

For both translation models, the respective BLEU scores [39] were calculated on the shared 2016 Biomedical Translation Task [27]. The chosen BLEU algorithm was the weighted geometric mean of the n-gram precisions per sentence.

A fine-tuned version of OPUS-MT-FR-EN [16] was also tested on the 2016 and 2019 Biomedical Translation shared tasks. For fine-tuning, we used the following hyperparameters: a maximum sequence length of 128 (mainly for computational memory reasons), a learning rate of 2 × 10^–5^, and a weight decay of 0.01, and we varied the number of epochs up to 15 epochs (the error function curve stops decaying after 10 epochs). The Google Translate model could not be used for our clinical score experiments for reasons of confidentiality.

Table 5 presents the BLEU scores for the 3 models, showing that fine-tuning the OPUS-MT-FR-EN model [16] on biomedical data sets gave the best results, with a BLEU score [39] of 0.51. This was the model used to calculate the overall performance of axes 2.1 and 2.2.

**Table 5.** Translation performances: BLEU scores of the translation models. The best performance value is italicized.

Models	WMT^a^ Biomed 2016 test
Google Translate	0.42
OPUS-MT-FR-EN	0.31
OPUS-MT-FR-EN FT^b^	*0.51*

a WMT: Workshop on Machine Translation.

b OPUS-MT-FR-EN FT corresponds to the OPUS-MT-FR-EN model [16] *fine-tuned* on biomedical translated corpus from the WMT Biomedical Translation Tasks in 2016 [27] and 2019 [28].

### Overall Performances From Raw Text to CUI Predictions

This section presents the overall performance of the 3 axes, in an end-to-end pipeline. For axis 2, the results are those obtained with the best normalization algorithm (presented in Table 3). The model used for translation is the OPUS-MT-FR-EN [16] fine-tuned model. The results are presented in Table 4, with the best results obtained by the native French approach on the EMEA corpus [25] and French clinical notes. The 95% CIs were calculated using the empirical bootstrap method [40].

## Discussion

### Principal Findings

In this paper, we compared 2 approaches for extracting medical concepts from clinical notes: a French approach based on a French language model and a translated English approach, where we compared 2 state-of-the-art English biomedical language models, after a translation step. The main advantages of our experiment are that it is reproducible and that we were able to analyze the performance of each step of the algorithm: NER, normalization, and translation, and to test several models for each step.

### The Quality of the Translation Is Not Sufficient

We showed that the native French approach outperformed the 2 translated English approaches, even with a small French training data set. This analysis confirms that, where possible, an annotated data set improves feature extraction. The evaluation of each intermediate step showed that the performance of each module was similar in French and English. We can therefore conclude that it is rather the translation phase itself that is of insufficient quality to allow the use of English as a proxy without a loss of performance. This is confirmed by the translation performance calculations, where the calculated BLEU scores were relatively low, although improved by a fine-tuning step.

In conclusion, although translation is commonly used for entity extraction or term normalization in languages other than English [5,20,41-43], due to the availability of turnkey models that do not require additional annotation by a clinician, we showed that this induces a significant performance loss.

Commercial application programming interface–based translation services could not be used for our task due to data confidentiality issues. However, the OPUS-MT model is considered state of the art, it is adjustable to domain-specific data, and the translation results presented in Table 5 confirm the absence of performance difference between this model and the Google Translate model.

Although our experiments were carried out on a single language, the French-English pair is one of the best performers in recent translation benchmarks [16]. Other languages are unlikely to produce significantly better results.

### Error Analysis

In these experiments, the overall results may appear low, but the task is still complex, especially because the UMLS Metathesaurus [1] contains many synonyms with different CUIs. To better understand this, we performed an error analysis on the normalization task only, as shown in Table S3 in Multimedia Appendix 1, with a physician’s evaluation, on a sample of 100 errors for both models. We calculated that 24% (24/100) and 39% (39/100) of the terms found by the deep normalization algorithm [14] and CODER [15], respectively, were in fact synonyms but had 2 different UMLS CUIs. This highlights the difficulty of achieving normalization on the UMLS Metathesaurus. The UMLS Metathesaurus indeed groups together numerous terminologies whose mapping between terms is often imperfect, implying that certain synonyms, as shown here, do not have the same CUI, as pointed out by Cimino [44] and Jiménez-Ruiz et al [45]. For example, “cardiac ultrasound” has the CUI of C1655737, whereas “echocardiography” has another CUI of C0013516; similarly, “H/O: thromboembolism” has a CUI of C0455533, whereas “history of thromboembolism” has a CUI of C1997787, and so on.

Moreover, to be more precise, each axis had its own errors: overall, the errors in axis 2 were essentially due to the loss of information in translation. One notable error was literal translation: for example, “dispersed lupus erythematous” instead of “systemic lupus erythematosus,” or “crepitant” instead of “crackles.” This loss of translation led to more errors in the extraction of named entities.

In addition to the loss of translation information, axis 2.1 was also penalized by the NER step, due to the difference between the training set (N2C2 notes) and the test set (the translated French notes; the aim being to compare the performance of English-language turnkey models with the performance of French-language models from an annotated set). Axis 2.1, for example, omitted the names of certain drugs more often.

Finally, both axes were penalized by abbreviations. These were often badly translated (for example, the abbreviation “MFIU” for “mort foetale in utero,” meaning “intrauterine fetal death,” was not translated), which penalized axis 2. Nevertheless, if they were indeed extracted by NER steps in axis 1, they were not correctly normalized due to the absence of a corresponding CUI in the UMLS Metathesaurus.

### Limitations

This work has several limitations. First, the actual French clinical notes contained very few terms in the *Devices* semantic group, which prevented the NER algorithm from finding them in the test data set. However, this drawback, which penalized the native French approach, still allowed us to draw a conclusion for the results. Furthermore, in this study, we did not take into account attributes of the extracted terms such as negation, hypothetical attribute, or belonging to a person other than the patient for comparison purposes, as the QUAERO [25] and N2C2 2019 [24] data sets did not have this labeled information.

## Supplementary material

10.2196/49607 Multimedia Appendix 1 Detailed description of the data sets, an example of the clinical notes annotation, French corpus annotation, MedCAT performances, and error analysis.

## Abbreviations

BERT: Bidirectional Encoder Representations From Transformers
CUI: concept unique identifier
N2C2: National Natural Language Processing Clinical Challenges
NER: named entity recognition
NLP: natural language processing
UMLS: United Medical Language System

## References

[1] Bodenreider O (2004). The Unified Medical Language System (UMLS): integrating biomedical terminology. Nucleic Acids Res.

[2] Vaswani A, Shazeer N, Parmar N, Guyon I, von Luxburg U, Bengio S (2017). Advances in Neural Information Processing Systems 30 (NIPS 2017).

[3] Devlin J, Chang MW, Lee K, Toutanova K, Burstein J, Doran C, Solorio T (2019). Proceedings of the 2019 Conference of the North American Chapter of the Association for Computational Linguistics: Human Language Technologies, Volume 1 (Long and Short Papers).

[4] Névéol A, Dalianis H, Velupillai S, Savova G, Zweigenbaum P (2018). Clinical natural language processing in languages other than English: opportunities and challenges. J Biomed Semantics.

[5] van Mulligen EM, Afzal Z, Akhondi SA, Vo D, Kors JA, Balog K, Cappellato L, Ferro N, Macdonald C (2016). Working Notes of CLEF 2016 - Conference and Labs of the Evaluation Forum CEUR Workshop Proceedings, Vol 1609.

[6] Gao Q, Vogel S, Cohen KB, Carpenter B (2008). SETQA-NLP ’08: Software Engineering, Testing, and Quality Assurance for Natural Language Processing.

[7] Vogel S, Ney H, Tillmann C (1996). COLING ’96: Proceedings of the 16th Conference on Computational Linguistics - Volume 2.

[8] ChristelDG/biomed_translation. GitHub.

[9] Johnson AEW, Pollard TJ, Shen L (2016). MIMIC-III, a freely accessible critical care database. Sci Data.

[10] Lee J, Yoon W, Kim S (2020). BioBERT: a pre-trained biomedical language representation model for biomedical text mining. Bioinformatics.

[11] Huang K, Altosaar J, Ranganath R (2019). ClinicalBERT: modeling clinical notes and predicting hospital readmission. arXiv.

[12] Martin L, Muller B, Ortiz Suárez PJ, Kurafsky D, Chai J, Schluter N, Tetreault J (2020). Proceedings of the 58th Annual Meeting of the Association for Computational Linguistics.

[13] Le H, Vial L, Frej J, Calzolari N, Béchet F, Blanche P (2020). Proceedings of the Twelfth Language Resources and Evaluation Conference.

[14] Wajsbürt P, Sarfati A, Tannier X (2021). Medical concept normalization in French using multilingual terminologies and contextual embeddings. J Biomed Inform.

[15] Yuan Z, Zhao Z, Sun H, Li J, Wang F, Yu S (2022). CODER: knowledge-infused cross-lingual medical term embedding for term normalization. J Biomed Inform.

[16] Tiedemann J, Thottingal S, Martins A, Moniz H, Fumega S (2020). Proceedings of the 22nd Annual Conference of the European Association for Machine Translation.

[17] Zhang Y, Chen Q, Yang Z, Lin H, Lu Z (2019). BioWordVec, improving biomedical word embeddings with subword information and MeSH. Sci Data.

[18] Kraljevic Z, Bean D, Mascio A (2019). MedCAT -- medical concept annotation tool. arXiv.

[19] Campos L, Pedro V, Couto F (2017). Impact of translation on named-entity recognition in radiology texts. Database (Oxford).

[20] Suarez-Paniagua V, Dong H, Casey A, Faggioli G, Ferro N, Joly A, Maistro M, Piroi F (2021). Proceedings of the Working Notes of CLEF 2021 - Conference and Labs of the Evaluation Forum. CEUR Workshop Proceedings, Vol 2936.

[21] Perez-Miguel N, Cuadros M, Rigau G, Calzolari N, Choukri K, Cieri C (2018). Proceedings of the Eleventh International Conference on Language Resources and Evaluation (LREC 2018).

[22] Chen Y, Zong C, Su KYS, Hajič J, Carberry S, Clark S, Nivre J (2010). Proceedings of the 48th Annual Meeting of the Association for Computational Linguistics.

[23] Chen Y, Zong C, Su KYS (2013). A joint model to identify and align bilingual named entities. Comput Linguist.

[24] Henry S, Wang Y, Shen F, Uzuner O (2020). The 2019 National Natural Language Processing (NLP) Clinical Challenges (N2C2)/Open Health NLP (OHNLP) shared task on clinical concept normalization for clinical records. J Am Med Inform Assoc.

[25] Névéol A, Grouin C, Leixa J, Rosset S, Zweigenbaum P The QUAERO French medical corpus: a resource for medical entity recognition and normalization. https://perso.limsi.fr/pz/FTPapiers/Neveol_BIOTEXTM2014.pdf.

[26] Kors JA, Clematide S, Akhondi SA, van Mulligen EM, Rebholz-Schuhmann D (2015). A multilingual gold-standard corpus for biomedical concept recognition: the Mantra GSC. J Am Med Inform Assoc.

[27] Bojar O, Chatterjee R, Federmann C, Bojar O, Buck C, Chatterjee R (2016). Proceedings of the First Conference on Machine Translation: Volume 2, Shared Task Papers.

[28] Bawden R, Bretonnel Cohen K, Grozea C, Bojar O, Chatterjee R, Federmann C (2019). Proceedings of the Fourth Conference on Machine Translation (Volume 3: Shared Task Papers, Day 2).

[29] Wajsbürt P (2021). Extraction and Normalization of Simple and Structured Entities in Medical Documents [thesis].

[30] Gérardin C, Wajsbürt P, Vaillant P, Bellamine A, Carrat F, Tannier X (2022). Multilabel classification of medical concepts for patient clinical profile identification. Artif Intell Med.

[31] Lample G, Ballesteros M, Subramanian S, Kawakami K, Dyer C, Knight K, Nenkova A, Rambow O (2016). Proceedings of the 2016 Conference of the North American Chapter of the Association for Computational Linguistics: Human Language Technologies.

[32] Hochreiter S, Schmidhuber J (1997). Long short-term memory. Neural Comput.

[33] Kim J, El-Khamy M, Lee J (2017). Residual LSTM: design of a deep recurrent architecture for distant speech recognition.

[34] Yu J, Bohnet B, Poesio M, Jurafsky D, Chai J, Schulter N, Tetreault J (2020). Proceedings of the 58th Annual Meeting of the Association for Computational Linguistics.

[35] Kingma DP, Ba J (2014). Adam: a method for stochastic optimization. arXiv.

[36] Bojanowski P, Grave E, Joulin A, Mikolov T (2017). Enriching word vectors with subword information. Trans Assoc Comput Linguist.

[37] Wang X, Han X, Huang W, Dong D, Scott MR (2019). Multi-similarity loss with general pair weighting for deep metric learning.

[38] CNIL (Commission Nationale de l’Informatique et des Libertés).

[39] Papineni K, Roukos S, Ward T, Zhu W-J (2002). ACL ’02: Proceedings of the 40th Annual Meeting on Association for Computational Linguistics.

[40] Dekking FM, Kraaikamp C, Lopuhaa HP, Meester LE (2007). A Modern Introduction to Probability and Statistics: Understanding Why and How.

[41] Cotik V, Rodríguez H, Vivaldi J, Lossio-Ventura J, Muñante D, Alatrista-Salas H Information Management and Big Data. SIMBig 2018. Communications in Computer and Information Science, vol 898.

[42] Hellrich J, Hahn U, Métais E, Roche M, Teisseire M (2014). Natural Language Processing and Information Systems. NLDB 2014. Lecture Notes in Computer Science, vol 8455.

[43] Attardi G, Buzzelli A, Sartiano D, Forner P, Navigli R, Tufis D, Ferro N (2013). Working Notes for CLEF 2013 Conference. CEUR Workshop Proceedings, Vol 1179.

[44] Cimino JJ (1998). Auditing the Unified Medical Language System with semantic methods. J Am Med Inform Assoc.

[45] Jiménez-Ruiz E, Grau BC, Horrocks I, Berlanga R (2011). Logic-based assessment of the compatibility of UMLS ontology sources. J Biomed Semantics.

[46] Assistance Publique Hôpitaux de Paris.

