# Supplementary material for: Impact of Translation on Biomedical Information Extraction: Experiment on Real-Life Clinical Notes
Source: JMIR Med Inform. 2024 Apr 4;12:e49607. doi: 10.2196/49607 (PMC11007378; doi:10.2196/49607)
Supplement: Multimedia Appendix 1 [file medinform-v12-e49607-s001.docx]

**Multimedia Appendix 1**

**Table S1.** **Datasets.** Detailed description of the size of the datasets with the number of annotated entities (with UMLS semantic groups and CUIs).

| Datasets  Types | QUAERO | | French  notes | n2c2  2019 | Mantra  English |
| --- | --- | --- | --- | --- | --- |
|  | EMEA | MEDLINE |  |  |  |
| CHEM | 2481 | 1053 | 869 | 1556 | 260 |
| DEVI | 170 | 128 | 57 | 193 | 11 |
| DISO | 1509 | 2837 | 2617 | 4463 | 341 |
| PROC | 952 | 1749 | 2310 | 3834 | 150 |
| Overall | 5112 | 5767 | 5853 | 10046 | 762 |


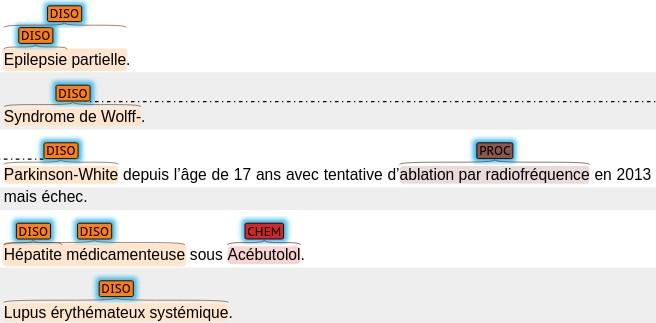


**Figure S1**: Example of clinical notes annotation with the four groups of interest (CHEM, DEVI, DISO, PROC)

**Section S1**. French corpus annotation

Semantic group and CUI annotation on clinical notes was performed by a physician. A total of 42 hospitalization reports from different departments: internal medicine, gynecology, rheumatology, etc. were annotated corresponding to a total of 5853 entities, as shown in the Supplementary Table 1. All corresponding CHEM, DEVI, DISO and PROC entities were annotated, including the case where the concept was not present in the UMLS^®^ (neither in French nor in English). Figure 3 gives an example of this annotation with the four different groups. Following QUAERO[25] annotation rules, nested entities were also annotated. The section on data availability below details the conditions for sharing this corpus.

**Table S2. MedCAT performances.** Presentation of the overall results of MedCAT (NER + normalization), focusing on the four semantic groups of interest : CHEM, DEVI, DISO, PROC.

|  | n2c2 | 2019 | test | Mantra English | | |
| --- | --- | --- | --- | --- | --- | --- |
| MedCAT | precision | recall | f1-score | precision | recall | f1-score |
|  | 0.41 | 0.54 | 0.46 | 0.52 | 0.47 | 0.48 |

**Table S3.** **Error analysis for the normalization step.** This table presents a list of normalization errors made by the deep multilingual model described in section 3.5.1, terms with very similar meaning or synonyms are highlighted in orange.

| true text mention | **CUI pred** | **UMLS term** | **CUI stand** | **UMLS term** |
| --- | --- | --- | --- | --- |
| fcs | C0587248 | FCS-304 | C0000786 | Spontaneous abortion |
| hb | C0019046 | Hemoglobin | C0518015 | Hemoglobin measurement |
| transfusion | C1879316 | Transfusion (procedure) | C0005841 | Blood Transfusion |
| fibrinogène | C0337428 | Fibrinogen assay | C2065007 | fibrinogen transfusion |
| bilan biologique | C2717898 | Biostatistics | C1511148 | Biological Testing |
| ionogramme | C0288925 | Ionogran | C0853360 | Blood electrolytes |
| ecg | C1623258 | Electrocardiography | C0013798 | Electrocardiogram |
| hospitalisée | C0701159 | Patient in hospital (finding) | C0019993 | Hospitalization |
| rai | C3178806 | Right Atrial Isomerism | C0430262 | Indirect Coombs Test |
| sleeve | C0878989 | Conductive Sleeves | C4758637 | Gastric sleeve |
| péridurale | C1283259 | Epidural device | C0002913 | Epidural Anesthesia |
| curetage | C0180236 | Curette | C0010468 | Dilatation and Curettage |
| remplissage | C0035139 | Surgical Replantation | C4761258 | Blood volume expansion |
| pfc | C0070493 | PFL protocol | C0016709 | Fresh frozen plasma |
| noradrénaline | C0202145 | Norepinephrine measurement | C0028351 | norepinephrine |
| artério-embolisation | C3163695 | Transarterial embolization | C0397760 | Embolization of artery |
| nacl | C0037494 | sodium chloride | C0036082 | Saline Solution |
| kta | C0313203 | Blood group antibody EnˆaˆKT | C0581447 | Arterial catheter |
| morphine | C2698261 | Morphine Measurement | C0026549 | morphine |
| rl | C2935745 | yellow RL | C0073385 | Lactated Ringer’s Solution |
| rx | C0809849 | Radiation Rx | C1306645 | Plain x-ray |
| normotendue | C0606030 | Normoten | C2712122 | Normal blood pressure |
| calcémie corrigée  normale | C3468265 | CALCR normal variant | C0853771 | Blood calcium normal |
| troponine  en  diminution | C0041199 | Troponin | C5442263 | Troponin decreased |
| examen clinique | C1456356 | examination; clinical | C0031809 | Physical Examination |
| imagerie | C0441633 | Scanning | C0011923 | Diagnostic Imaging |
| thromboemboliques | C0016018 | Fibrinolytic Agents | C0040038 | Thromboembolism |
| sigmoïdites  diverticulaires | C1395719 | sigmoid; diverticulum | C0012814 | Colonic Diverticulitis |
| histologique | C0344441 | Histology Procedure | C0679557 | histological diagnosis |
